# Identification of Novel *Trypanosoma cruzi* Cysteine Protease Inhibitors via Ligand-Based Virtual Screening of FDA-Approved Drugs with Trypanocidal Activity

**DOI:** 10.3390/diseases14020079

**Published:** 2026-02-19

**Authors:** Lenci K. Vázquez-Jiménez, Alonzo González-González, Timoteo Delgado-Maldonado, Rogelio Gómez-Escobedo, Benjamín Nogueda-Torres, Ana Verónica Martínez-Vázquez, Eyrá Ortiz-Pérez, Charmina Aguirre-Alvarado, Verónica Alcántara-Farfán, Joaquín Cordero-Martínez, Lorena Rodríguez-Páez, Adriana Moreno-Rodriguez, Gildardo Rivera

**Affiliations:** 1 Laboratorio de Biotecnología Farmacéutica, Centro de Biotecnología Genómica, Instituto Politécnico Nacional, Reynosa 88710, Mexico; lenka.18@hotmail.com (L.K.V.-J.); al.gonzalez.gonzalez88@gmail.com (A.G.-G.); titi_999@live.com (T.D.-M.); avmartinez@ipn.mx (A.V.M.-V.); eortizp@ipn.mx (E.O.-P.); 2 Secretaría de Ciencia, Humanidades, Tecnología e Innovación (SECIHTI), Ciudad de Mexico 03940, Mexico; 3 Departamento de Parasitología, Escuela Nacional de Ciencias Biológicas, Instituto Politécnico Nacional, Ciudad de México 11340, Mexico; rogelio.gomez.14@gmail.com (R.G.-E.); bnogueda@ipn.mx (B.N.-T.); 4 Departamento de Bioquímica, Escuela Nacional de Ciencias Biológicas, Instituto Politécnico Nacional, Ciudad de México 11340, Mexico; caguirrea@ipn.mx (C.A.-A.); valcantaraf@ipn.mx (V.A.-F.); jcorderom@ipn.mx (J.C.-M.); lrodrig@ipn.mx (L.R.-P.); 5 Laboratorio de Estudios Epidemiológicos, Clínicos, Diseños Experimentales e Investigación, Facultad de Ciencias Químicas, Universidad Autónoma “Benito Juárez” de Oaxaca, Avenida Universidad S/N, Ex Hacienda Cinco Señores, Oaxaca 68120, Mexico; arimor10@hotmail.com

**Keywords:** *Trypanosoma cruzi*, cruzain, drug repositioning, FDA drugs, enzyme inhibition

## Abstract

Background: Chagas disease is a major public health problem, especially in Latin American countries, and benznidazole and nifurtimox are currently the only drugs available for its treatment. However, they present several disadvantages, such as low availability, high toxicity, and limited efficacy, which often result in treatment discontinuation. In recent decades, bioinformatics studies have accelerated the field of drug repurposing, reducing time and costs. In this study, the aim was to identify novel cruzain inhibitors from the analogs of FDA-approved drugs with trypanocidal activity. Methods: A ligand-based virtual screen, along with molecular docking analysis, was carried out, and the selected compounds were evaluated for their trypanocidal activity against trypomastigotes of two endemic Mexican strains and their inhibitory activity on cysteine proteases. Results: A cefsulodin analog (LC_50_ = 126.18 and 77.50 µM), two flucloxacillin analogs (LC_50_ = 94.05 and 101.73 µM; 48.74 and 64.49 µM), and one piperacillin analog (LC_50_ = 48.46 and 83.68 µM) had better trypanocidal activity and selectivity index against the NINOA and INC-5 strains than the reference drugs. Enzymatic evaluation showed that all four compounds inhibited cysteine proteases (IC_50_ < 840.03 µM). Furthermore, molecular dynamics simulations predicted the stability of the compound–protein complex, while the docking test on human cathepsin L predicted their potential selectivity. Finally, our in silico analysis of ADMET properties showed that all compounds exhibited favorable profiles. Conclusions: These results encourage the development of new and more potent anti-*Trypanosoma cruzi* agents using FDA-approved drugs as scaffolds.

## 1. Introduction

Chagas disease, which is caused by the parasite *Trypanosoma cruzi* (*T. cruzi*), is one of the leading causes of parasitic disease-associated mortality in Latin America. According to the World Health Organization (WHO), approximately 7 million people worldwide are currently infected [1]. Specifically, approximately 1 million individuals in Mexico are infected, with another 29.5 million at risk of being infected [2]. To date, the current chemotherapeutic treatment is based on the drugs nifurtimox and benznidazole. However, its efficacy is limited and variable (60–80% in the acute phase and 20–40% in the chronic phase), causing severe adverse effects that promote treatment abandonment by the patients [3,4]. Therefore, there is a need to develop new, more effective, and less cytotoxic therapeutic options. In this regard, an alternative is the identification of molecules targeting established pharmacological targets in this parasite, such as cruzain (Cz), a cysteine protease that plays an important role in the life cycle of *T. cruzi*, including metacyclogenesis and immune evasion [5].

On the other hand, there are several approaches to novel drug development; however, most of them are costly and time-consuming. However, one method, Computer-Aided Drug Design (CADD), has stood out in recent decades due to its low costs and shorter development time, increasing the odds of novel drug discovery. This alternative has enabled the easier identification of molecules with “new activity”, including those approved by regulatory agencies, thus leading to drug repositioning [6].

**Figure 1:**
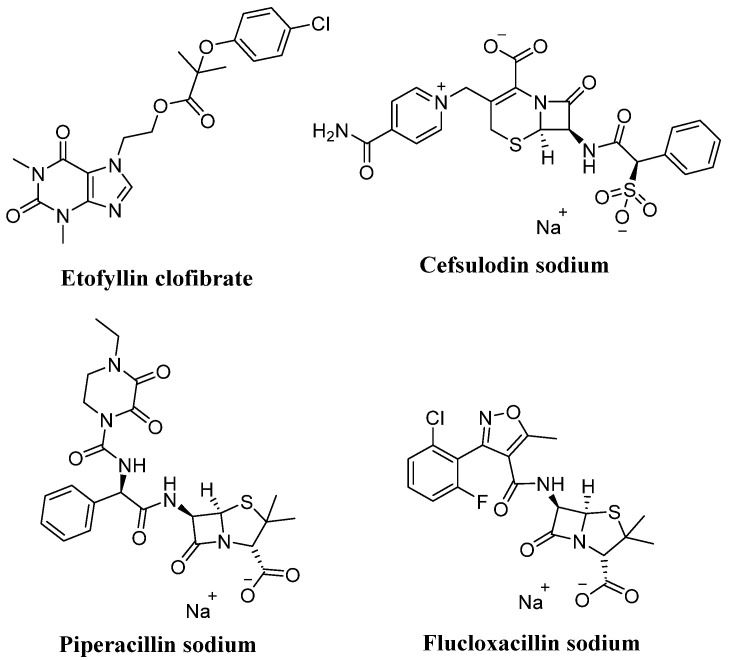
Chemical structures of repurposed FDA-approved drugs for the potential treatment of Chagas disease.

Based on the above information and considering *T. cruzi* Cz (*Tc*Cz) as a pharmacological target, as well as the use of CADD strategies and their subsequent validation in *in vitro* and *in vivo* models, Palos et al. identified four drugs (etofylline, cefsulodin, flucoxacillin, and piperacillin) with trypanocidal activity that are emerging as possible therapeutic candidates for the treatment of Chagas disease (Figure 1) [7]. Therefore, the structures of these four FDA-approved drugs were used in this study to identify potential Cz inhibitors in the MolPort chemical library via ligand- and molecular docking-based virtual screening. Subsequently, the most promising candidates were evaluated *in vitro* against blood trypomastigotes from two strains of *T. cruzi* and against the parasite’s cysteine proteases to confirm their potential mechanism of action. Finally, molecular dynamics analysis was performed to predict their stability when complexed with a protein, and further molecular docking studies were conducted to estimate their potential selectivity for human cathepsin L (*Hs*CatL), a cysteine protease homologous to *T. cruzi*.

## 2. Materials and Methods

### 2.1. Protein Preparation

The crystal structure of *Tc*Cz in complex with the co-crystallized ligand *N*-(1*H*-benzimidazol-2-yl)-1,3-dimethyl-1*H*-pyrazole-4-carboxamide (3H5) was obtained from the Protein Data Bank (PDB) database (https://www.rcsb.org/) using accession code 4W5B [7], and that of human cathepsin L (*Hs*CatL) was retrieved from the AlphaFold web server using Uniprot ID P07711. Their crystal structures were generated using the open-access software UCSF-Chimera 1.15 [8], removing all co-crystallized molecules and employing the built-in Dock Prep tool to add hydrogen atoms and partial Gasteiger charges to the proteins. The prepared proteins were saved in .pdb format.

### 2.2. Ligand Library Preparation

Three ligand libraries were obtained from the MolPort chemical database by performing a search of the reused parent ligands while considering a 50% Tanimoto similarity score: flucloxacillin, etofylline, cefsulodin, and piperacillin. The search was limited to commercially available compounds in the database, and each exported ligand file in .sdf format was converted to .smi format, then cleaned of any additional salts or molecules included in the initial sdf file but not part of the parent ligand. The cleaned .smi file was imported and filtered using Data Warrior software, with Lipinski and Veber rules serving as the inclusion criteria for the selected compounds. These compounds were then exported as a 2D sdf file. These libraries were then further filtered to detect any repeated molecules, and 3D energy minimization was performed using the Universal Force Field (UFF). The resulting structures were then exported in .sdf format using the open-access software OpenBabel 3.1 [9].

### 2.3. Molecular Docking

The docking software gnina version 1.0.2 [10] was used to perform standard molecular docking simulations for each chemical library obtained from Molport, as well as for reference ligands co-crystallized with ligand 3H5. The simulations were carried out considering a cubic grid box centered at the coordinates corresponding to the center of mass for the co-crystallized ligand for each respective protein. The grid box was configured with an edge length of 24 Å for each XYZ dimension using a spacing of 1 Å, totaling 13,824 grid points. The *Hs*CatL receptor was 3D-aligned to the *Tc*Cz receptor (RMSD 1.82 Å), and the coordinates utilized for the parasite protein were maintained for the human protein.

### 2.4. Molecular Docking Analyses

The molecular docking results were analyzed under the criteria based on their binding free energy (BFE) and amino acid residue interaction profiles. Initially, ligands were filtered by their BFE values compared to the reference ligand (3H5), and only compounds with higher affinity than ligand 3H5 were selected for further analysis. Next, compounds that met the energy criterion were evaluated through their interaction profile using the Protein–Ligand Interaction Profiler (PLIP) version 2.2.2 software [11]. This profile served as a fingerprint, which was then compared to that of the reference ligand. The Python version 3.9 software package sklearn was used to calculate the Jaccard distance between fingerprints, generating a distance matrix for each library. The Euclidean metrics from the K-means clustering function in sklearn were applied to the distance matrix to determine distortion and inertia measurements, which were plotted to identify the optimal number of clusters using the “elbow method” [12]. Finally, the data were clustered into N groups, and the top ten compounds with the best BFE values, as well as grouped with the 3H5 ligands, were considered for purchase and in vitro studies.

### 2.5. Trypanocidal Assay

Six- to eight-week-old CD1 mice infected with the blood trypomastigotes of the INC-5 and NINOA strains were used. At peak parasitemia (2 to 4 weeks), parasitized blood was collected via cardiac puncture, with sodium heparin used as the anticoagulant. The blood concentration was adjusted to 1 × 10^6^ trypomastigotes/mL. Ninety microliters of infected blood and 10 µL of the selected compounds (dissolved in 1% DMSO) were added to a 96-well plate, yielding a final volume of 100 µL per well. Benznidazole and nifurtimox were used as positive controls, and untreated blood trypomastigote wells served as the negative control. The microplates were incubated at 4 °C for 24 h. To quantify motile trypomastigotes, 5 μL of blood was placed on a slide and covered with an 18 × 18 mm coverslip, and motile protozoa were counted in 15 fields at 40× magnification using a light microscope. The lysis percentage for each treatment was calculated by comparing the viable trypomastigotes with the negative control [13]. The mean lytic concentration (LC_50_) was determined for each compound using Probit statistical analysis, and the assay was performed in triplicate. The compounds analyzed were purchased from the commercial company MolPort and used without further purification. The MolPort codes for the compounds are as follows: Molport-009-696-999 (**AG-218**), Molport-038-939-984 (**AG-984**), Molport-009-697-132 (**AG-132**), Molport-009-696-988 (**AG-988**), Molport-002-666-405 (**AG-405**), Molport-009-738-615 (**AG-615**), Molport-009-479-287 (**AG-287**), Molport-007-908-410 (**AG-410**), Molport-001-567-525 (**AG-525**), Molport-005-628-899 (**CZ-899**), Molport-019-797-018 (**CZ-018**), Molport-009-197-595 (**AG-595**), and Molport-005-061-173 (**CZ-173**).

### 2.6. Cytotoxicity Assay in Murine Macrophages

Cytotoxicity assays were performed using the mouse macrophage cell line (J774.2). Specifically, cells were cultured in an RPMI medium supplemented with 10% SBF, penicillin (100 µg/mL), streptomycin (100 µg/mL), and glutamine (2 mM) in a 5% CO_2_ atmosphere, with the culture medium being replaced every 2 to 3 days, and macrophages were used at a concentration of 1 × 10^−6^ cells and incubated with the compounds at concentrations of 0.75 to 100 µM at 37 °C for 48 h in a 5% CO_2_ atmosphere. A 0.2% DMSO solution was used as the negative control, while the reference drugs were used as the positive control. Cellular metabolic activity and the half-maximal cytotoxic concentration (CC_50_) were determined using the MTT method and Probit analysis, respectively, and the cell viability percentage was also calculated [14]. The assays were performed in triplicate, and the selectivity index (SI) (CC_50_/CI_50_) was calculated.

### 2.7. Inhibition Assay of T. cruzi Cysteine Proteases

The protein extract was obtained from the epimastigotes of the *T. cruzi* strain NINOA and quantified using the Bradford method [15]. An activity assay was carried out using 5 µM of the fluorogenic substrate Z-Phe-Arg-MAC, 1 µg of protein extract, and concentrations of 0.01 to 1000 µg/mL of the tested compounds **AE-410**, **AG-615**, **CZ-018,** and **CZ-173,** following the methodology described by Santos et al., [16] and the assay was performed in a SpectraMax M5 spectrofluorometer (Molecular Devices, San Jose, CA, USA). The mean inhibitory concentration (IC_50_) values were determined from semi-logarithmic concentration–response curves obtained using nonlinear regression, which was performed using the Dose–Response Analysis (DRA) package of the R software (version 4.3.1) [17].

### 2.8. Molecular Dynamics

Molecular dynamics (MD) analysis was performed for the chosen compounds to analyze protein–ligand complex stability and thus assess their potential as stable *Tc*Cz inhibitors. The analysis was carried out using GROMACS version 2018.4 software [18] with a 200 ns final simulation at a temperature of 300 K [19,20]. The topology of each compound was generated in the Amber General Force Field of the ACPYPE Antechamber module, and water molecules were added to a dodecahedral box with a minimum distance of 10 Å from the walls, using the TIP3P water model for solvation. Subsequently, Na+/Cl− ions were added to neutralize the system, and energy minimization was performed 50,000 times using the “steepest descent” algorithm. Equilibrium was determined at 300 K in two phases: In the first phase, the ligand was simulated under NVT (number of particles, volume, and temperature) conditions using a V-rescale thermostat with a time constant (tau_t) of 0.1 ps, assigning velocities according to the Maxwell–Boltzmann distribution. In the second phase, the ligand was simulated under NPT conditions (a constant number of particles, 1 atm pressure, and a constant temperature) using a V-rescaling thermostat and a Berendsen barostat with time constants (tau_t and tau_p) of 0.1 and 2.0 ps, respectively. Each phase lasted 100 ps, and the stability of the complex was evaluated by calculating its root mean square deviation (RMSD), root mean square fluctuation (RMSF), and radius of gyration (Rgyr) using the GROMACS software tools [21].

### 2.9. In Silico Pharmacokinetic Analysis

The ADMETlab 2.0 website (https://admetmesh.scbdd.com/) [22] (accessed on 30 August 2024) was used to determine the physicochemical and pharmacokinetic properties of the selected compounds, while the ProTox-II server [23] (accessed on 27 August 2024) was used to predict compound toxicity.

## 3. Results and Discussion

### 3.1. Protein–Ligand Analysis

**Figure 2:**
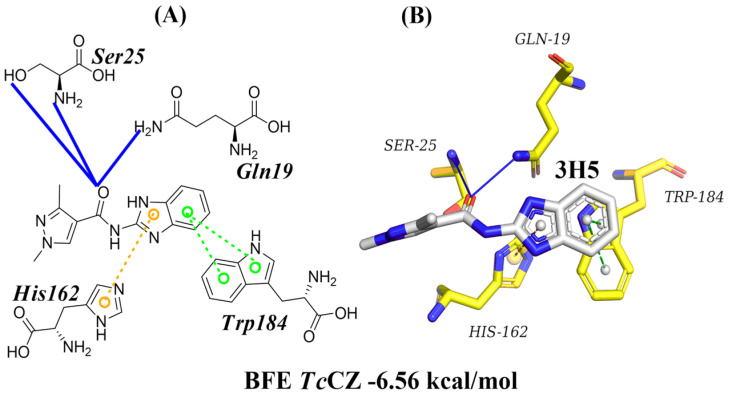
Molecular docking analysis of the 3H5 ligand co-crystallized with cruzain (4W5B) from *T. cruzi*. (**A**) A 2D image of the 3H5 ligand interaction profile. (**B**) A 3D image of the 3H5 ligand interaction profile. Hydrogen bonds are shown as blue lines, while cation–π interactions are shown as yellow dashed lines, and stacking–π interactions are displayed as green dashed lines.

The crystal *Tc*Cz protein with code 4W5B was retrieved from the PDB database (www.rcsb.org/structure/4W5B, accessed on 1 July 2024). This crystal has a resolution of 2.7 Å and co-crystallized with a known non-covalent inhibitor, 3H5. Initially, the molecular docking protocol of the *Tc*Cz protein with the co-crystallized ligand 3H5 was validated, and an RMSD value of 2.4 Å was obtained, which indicates that the docking pose deviates from the reference but maintains the same general orientation; an overlapped pose is shown in Figure S1 in the Supplementary Materials [24,25]. The 3H5 ligand had a BFE of −6.56 kcal/mol and interacted with four amino acid residues (GLN19, SER25, HIS162, and TRP184) on the active site of *Tc*Cz (Figure 2). These interactions are consistent with those described in the PDB. Furthermore, amino acid residues GLN19, SER25, HIS162, and TRP184 have been described as critical for this type of cysteine protease [26,27].

### 3.2. Ligand-Based Virtual Screening of the Molport Database

Considering four FDA-approved drugs (flucloxacillin, etofylline, cefsulodin, and piperacillin) as scaffolds, a virtual screening of the MolPort database was performed to obtain compounds with 50% structural similarity. Then, Lipinsky and Veber rules were applied, and finally, a molecular docking analysis was performed on the active site of *Tc*Cz. Subsequently, compounds with a better BFE than the control ligand (−6.56 kcal/mol) were selected, and all compounds that shared at least one interaction with the 3H5 ligand were selected and grouped. Table 1 shows the number of compounds obtained by applying each inclusion criterion. Finally, based on commercial availability, thirteen compounds were acquired for evaluation against *T. cruzi* trypomastigotes.

**Figure 3:**
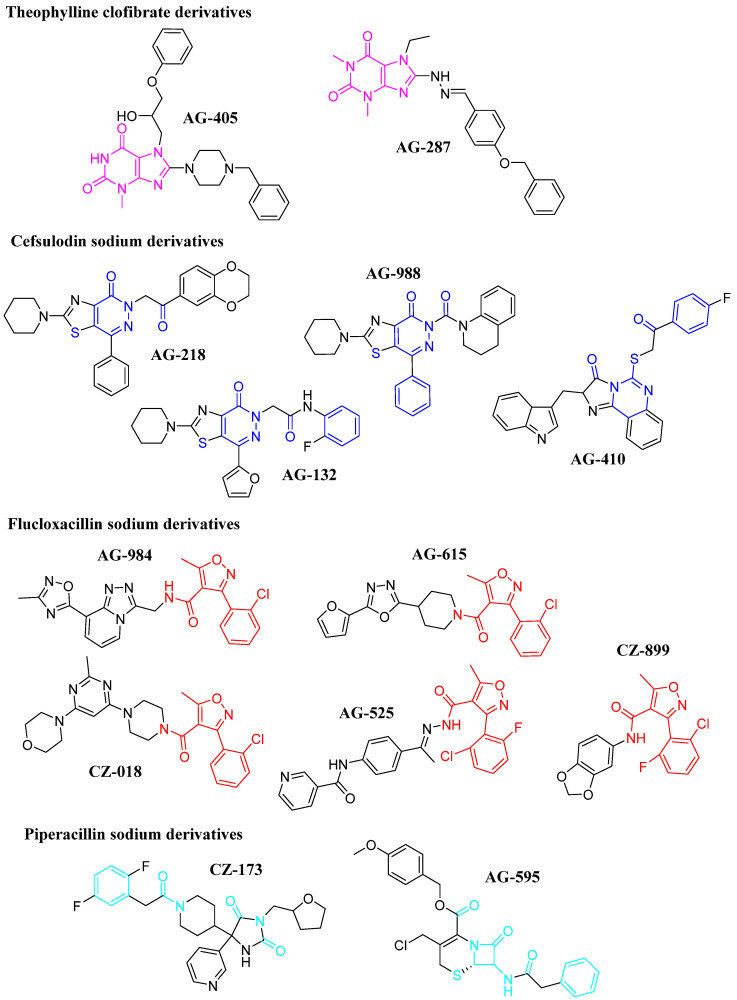
Chemical structures of the selected compounds as potential *Tc*Cz inhibitors. The colored structures are fragments of the structures shared with FDA-approved drugs with trypanocidal activity.

The chemical structures of the thirteen compounds, which were purchased commercially for the anti-trypanosomal assays due to their potential as *Tc*Cz inhibitors, are shown in Figure 3. These compounds represent the most promising candidates, while the others were discarded as they did not meet one of the established filters or were not commercially available.

Despite partially retaining the theophylline moiety in their structure, the theophylline derivatives **AG-405** and **AG-287** showed no activity against any of the evaluated strains. Regarding the cefsulodin derivatives, only compound **AG-410** exhibited activity against both strains, and this was the only member of this group that did not retain the pyridazine-3-one or sulfonyl group in the same position as the other compounds, suggesting that these structural modifications could be important for biological activity. In the case of the flucloxacillin derivatives, only **AG-615** and **CZ-018** showed trypanocidal activity against the NINOA and INC-5 strains. Both compounds share a pyridine ring, suggesting that this structural motif could contribute favorably to antiparasitic activity. Finally, of the two piperacillin derivatives evaluated, only **CZ-173** showed biological activity. Unlike **AG-595**, this compound lacks the β-lactam ring, the absence of which could reduce potential bacterial resistance mechanisms and contribute to the observed activity.

Overall, the results showed differences in trypanocidal potency among the evaluated derivatives. Compounds **AG-615**, **CZ-173**, **AG-410**, and **CZ-018** exhibited the lowest LC_50_ values against both analyzed strains, outperforming the reference drugs benznidazole and nifurtimox. In contrast, several analogs (**AG-287**, **AG-132**, **AG-984**, and **AG-525**) were inactive (LC_50_ > 200 µM), indicating that seemingly minor modifications to the substituents significantly impact their biological activity. This trend suggests that the most active compounds share structural characteristics that favor specific interactions with the molecular target, likely through a better balance between lipophilicity and polarity and a greater capacity to form hydrogen bonds. In addition, strain-dependent behavior was observed, as in the case of **AG-595**, which was only active against NINOA, potentially reflecting differences in cell permeability or protease expression between strains.

### 3.3. Molecular Docking Analyses

**Figure 4:**
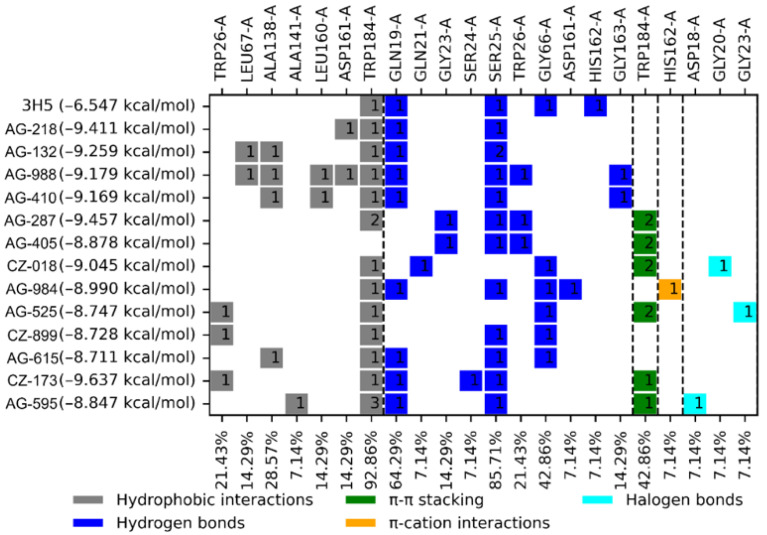
The interaction profile of the thirteen compounds selected as potential *Tc*Cz inhibitors and the control ligand 3H5. The number displayed inside the interaction box represents the number of interactions.

The BFE values and interaction profiles of the thirteen compounds selected as potential *Tc*Cz inhibitors are shown in Figure 4. These compounds showed BFE values between −9.63 and −8.71 kcal/mol, which were higher than that of the control ligand 3H5 (−6.54 kcal/mol). Compound **CZ-173** had the best BFE value (−9.63 kcal/mol). The compounds exhibited hydrophobic interactions, hydrogen bonds, halogen bonds, π-π stacking, and π–cation interactions. Compound **AG-988** showed the highest number of interactions (nine), followed by compound **AG-595** (eight). The control ligand (3H5) interacted with five amino acid residues: TRP184 via a hydrophobic interaction and GLN21, SER25, GLY66, and HIS162 via hydrogen bonds. Most of the compounds showed at least three of these interactions, and the most frequent interaction was with residue TRP184 (hydrophobic interactions in 93% of cases), followed by SER25 (85.71%) and GLN21 (64.29%) via hydrogen bonds. GLY66 (hydrogen bond) and TRP184 (π-π stacking) had a frequency of 42.86%.

### 3.4. Trypanocidal Activity

The thirteen selected compounds were evaluated against the trypomastigote form, which was selected due to its role in the acute phase of Chagas disease. The NINOA and INC-5 strains of *T. cruzi* were used for the evaluation (Table 2) (dose–response curves are available in Figures S2–S15). Nine compounds (**AG-988**, **AG-405**, **AG-218**, **AG-410**, **AG-615**, **CZ-018**, **CZ-899**, **AG-595**, and **CZ-173**) had better LC_50_ values than the reference drugs benznidazole and nifurtimox (LC_50_ = 161.05 and 219.82 µM, respectively) against one or both tested strains. **AG-595** and **CZ-173** had the highest LC_50_ values against the NINOA strain (LC_50_ = 25.54 and 48.46 µM, respectively), while **CZ-018** had the best LC_50_ value (64.49 µM) against INC-5. Compounds **AG-410**, **AG-615**, **CZ-018**, and **CZ-173** were more active than the reference drugs (LC_50_ = 254.93 and 317.85 µM, respectively) against both strains (Table 2).

### 3.5. Cytotoxicity Assay in Murine Macrophages

For cytotoxic evaluation against the J774.2 macrophage cell line, the four most active compounds against *T. cruzi* (**AG-410**, **AG-615**, **CZ-018**, and **CZ-173**) were selected. The results showed that all compounds had low cytotoxicity (CC_50_ > 236 µM) against the J774.2 cell line. In addition, the SI was determined to evaluate the relative selectivity of the compounds, including the reference drugs. All compounds showed higher SI values (SI > 2.36) than the reference drugs benznidazole and nifurtimox (SI < 0.83) against both strains analyzed (Table 3). It is worth noting that compound **CZ-018**, which exhibited the best trypanocidal activity against both strains (LC_50_ = 48.74 and 64.49 µM, respectively), also showed the best cytotoxicity (CC_50_ = 306.08 µM) and SI values (SI = 6.28 and 4.74, respectively). These results encourage further evaluation and indicate that these compounds are promising Chagas candidates.

### 3.6. Inhibition Assay of T. cruzi Cysteine Proteases

An enzymatic evaluation of compounds with better trypanocidal activity (**AG-410**, **AG-615**, **CZ-018**, and **CZ-173**) against *T. cruzi* cysteine proteases was carried out to confirm their mechanism of action (Table 4). The results showed that the four compounds selected presented enzymatic inhibition, with IC_50_ values from 44.0 to 840 µM. The cefsulodin derivative (**AG-410**) had an IC_50_ value of 120 µM, while the two flucloxacillin derivatives (**AG-615** and **CZ-018**) had contrasting values (840 and 85 µM, respectively), and the piperacillin derivative (**CZ-173**) was the most potent inhibitor with a value of 44 µM (Table 4). These results suggest that the compounds’ (**AG-410**, **CZ-018**, and **CZ-173)** trypanocidal activity is mediated through the inhibition of cysteine proteases. Interestingly, **CZ-173** showed an inhibitory value similar to that for Levothyroxine (IC_50_ = 38 µM), a *Tc*Cz inhibitor obtained via CAD repurposing [28]. Using the same strategy, clofazimine, benidipine, and saquinavir have been determined as *Tc*Cz inhibitors, with percentages of approximately 55 to 90% [29]. Other specific inhibitors obtained via the chemical synthesis of 1,4-naphthoquinone derivatives have inhibition values of 6.3 to 34.0 µM [30]. However, while the results indicate that the inhibition of cysteine proteases is part of the mechanism of action and considering that Cz constitutes the major fraction of the parasite’s proteolytic activity, the observed inhibition can be primarily attributed to this enzyme. Moreover, due to the use of the parasitic extract, the contribution of other minor cysteine proteases cannot be ruled out. Therefore, a specific study of the *Tc*Cz enzyme is still needed to accurately determine the type of inhibition exerted by these compounds.

Overall, nine compounded agents showed greater trypanocidal activity than benznidazole and nifurtimox against one or both strains of *T. cruzi*. Specifically, **CZ-018**, **AG-615**, **AG-410**, and **CZ-173** exhibited LC_50_ values between ~48 and 126 µM, while the reference drugs showed values of 161–317 µM under the same conditions. Furthermore, the most active compounds showed low cytotoxicity in macrophages (CC_50_ > 236 µM) and better SI (up to 6.28) compared to benznidazole and nifurtimox (SI < 0.83), with **CZ-018** standing out due to a combination of good antiparasitic potency (three times greater than benznidazole and four times greater than nifurtimox) and a more favorable safety profile (SI = 6.28). Although cysteine protease inhibition also occurs in the micromolar range, these values are comparable to those observed in previously reported *Tc*Cz inhibitors, as above-mentioned, and suggest that this mechanism contributes to the observed activity. Taken together, these results indicate an improvement over current drugs and position these compounds for further structural optimization aimed at increasing potency and broadening the therapeutic window.

### 3.7. Molecular Dynamics Analyses

**Figure 5:**
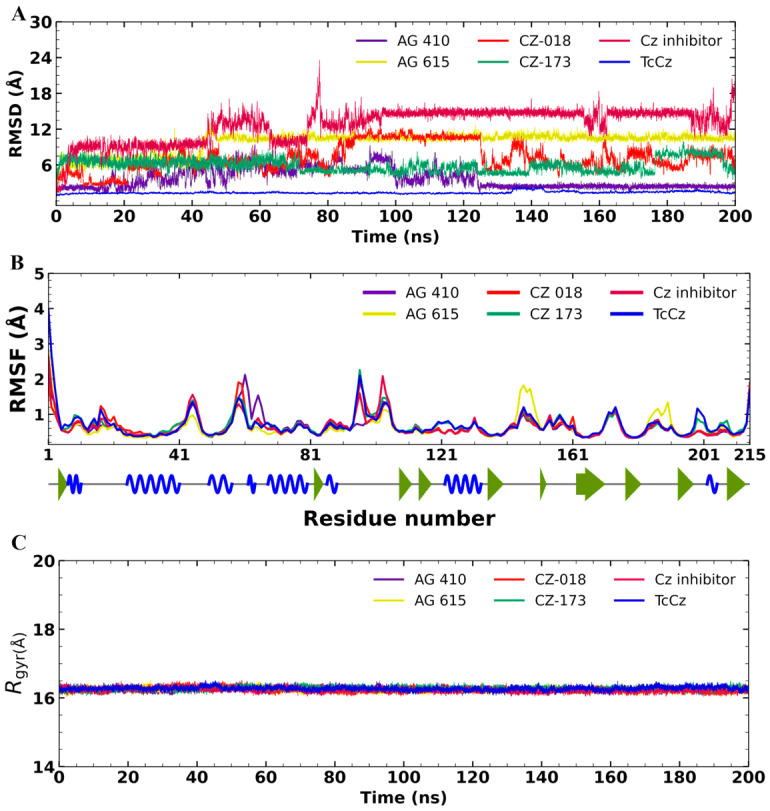
Analysis of the MD simulations of *Tc*Cz in complex with **AG-410**, **AG-615**, **CZ-018**, **CZ-173**, and the co-crystallized inhibitor 3H5. (**A**) The RMSD and (**B**) RMSF plots, where the green arrow indicates beta sheets, the alpha helix is depicted as a spiral blue line, and the gray line represents loops, as well as the (**C**) Rgyr plot.

Considering that the obtained compounds had trypanocidal activity and moderate inhibition of cysteine protease enzymes, MD simulations were performed to gain insight into their mechanism of action. The stability of each protein–ligand complex was evaluated at 200 ns. First, the apo form of *Tc*Cz was analyzed to determine the enzyme’s behavior in the absence of an inhibitor (3H5). The protein had an acceptable RMSD value (<2 Å), which validates that the system is adequate for evaluating its stability and does not observe fluctuations beyond those inherent to the protein (caused by the presence of small loops) (Figure 5A). The analysis of the MD trajectory for *Tc*Cz showed minimal fluctuations, with an average RMSD of 1.3 ± 0.2 Å.

On the other hand, the complex with the reference inhibitor 3H5 demonstrated stability during the first 45 ns; however, pronounced fluctuations were observed between 50 and 85 ns, and an acceptable behavior was subsequently observed, with an average RMSD of 12.7 ± 2.7 Å. These results indicate that the inhibitor 3H5 adopts a different orientation from its initial docking pose. This finding does not correlate with the actual co-crystallized complex due to covalent bonding. The ligand **AG-410** exhibited fluctuations of less than 6 Å during the first 100 ns; however, no fluctuations greater than 4 Å were observed for the remainder of the simulation. Hence, the global RMSD for **AG-410** was 3.6 ± 1.6 Å, which is considered stable.

The **AG-615**–*Tc*Cz complex had an average RMSD value of 9.7 ± 1.7 Å, and the trajectory analysis suggests that during the first 50s, **AG-615** adjusts within the protein cavity before reaching stability at the end of the MD simulation. These results suggest that the interactions of the *Tc*Cz–**AG-615** complex are not strong enough to obtain a pronounced effect.

The CZ-018 ligand showed several fluctuations throughout the MD simulation, with an average RMSD value of 7.1 ± 2.2 Å. However, noticeable stability was detected from 80 to 120 ns, correlating with moderate enzymatic inhibition. Finally, the ligand **CZ-173** exhibited minimal changes in terms of RMSD, with an average RMSD of 5.9 ± 1.1 Å. This value is lower than the previously reported threshold of <9 Å [31,32,33], indicating that this complex was stable during the analyzed period.

The RMSF is used to examine local changes along the protein chain and to characterize changes in ligand atom positions. In general, all RMSF calculations of ligands during their interaction with *Tc*Cz showed minimal fluctuations (Figure 5B). Ligands **AG-410** and **AG-615** and the reference inhibitor displayed some changes in the regions comprising residues 61–71, 141–151, and 181–191, respectively. However, these regions correspond to the protein’s loop regions, which are thought to mediate these oscillations. In summary, the fluctuations observed during the receptor–ligand interaction in the MD simulation were below 2.0 Å, which is considered acceptable and indicates that none of the ligands significantly perturbed the protein structure.

Figure 5C shows the Rgyr values for *Tc*Cz in apo-form and complex with each ligand. The mean Rgyr value for the apoprotein (blue) was 14.01 ± 0.05 Å. In all cases, the holoprotein in complex with each ligand exhibited an average Rgyr value of 13.98 ± 0.05 Å. These results indicate that the apo- and holo-forms of the protein remained stable throughout the 200 ns MD simulation. No significant differences were observed, suggesting that the protein maintains a compact structure during its dynamics.

Overall, the four compounds exhibited favorable docking scores and stable complexes during molecular dynamics simulations; however, compound **AG-615** had low enzymatic inhibition activity. This discrepancy can be attributed to the inherent limitations of molecular docking as a predictive tool. Docking scores primarily reflect static interactions and approximate affinity estimates without fully capturing solvation effects, entropic contributions, and conformational changes. Furthermore, molecular dynamics assesses complex stability but does not necessarily predict inhibitory capacity. Consequently, the computational approach was used as a prioritization strategy to enrich promising candidates, while experimental validation remains essential to confirm actual biological activity.

### 3.8. Molecular Docking on Human Homologs

**Figure 6:**
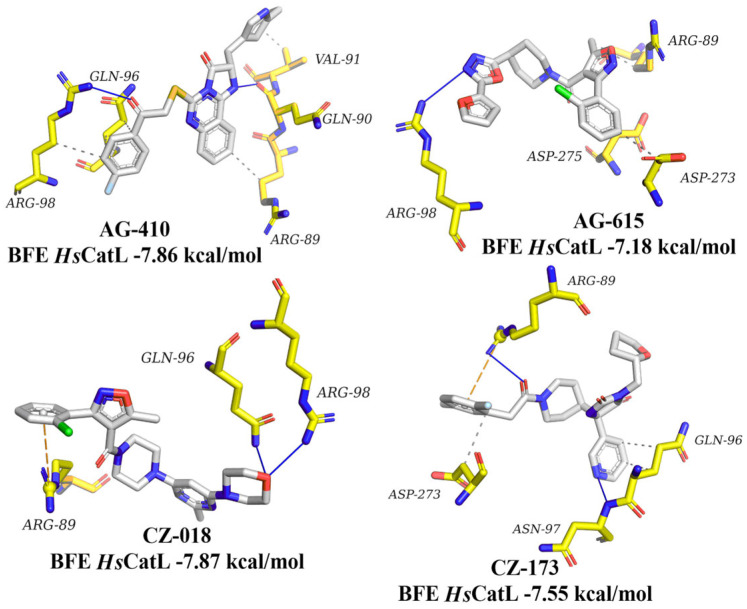
Interaction profile and BFE of **AG-410**, **AG-615**, **CZ-018**, and **CZ-173** on human cathepsin L. Hydrogen bonds are represented as blue lines, while hydrophobic interactions are displayed as a gray dashed line, and the π cation is represented as an orange dashed line.

Four compounds (**AG-410**, **AG-615**, **CZ-018**, and **CZ-173**) were evaluated via molecular docking on the active site of *Hs*CatL (Figure 6). These ligands showed lower affinity for *Hs*CatL, with BFE values ranging from −7.18 to −7.87 kcal/mol compared to those predicted for *Tc*Cz (−8.71 to −9.63 kcal/mol). Only 3 to 4 interactions were present per complex, and these mainly involved hydrophobic contacts and hydrogen bonds. Furthermore, if these interactions were rare, they were different from those predicted for *Tc*Cz. Therefore, based on the BFE and the interaction profile, the compounds may have selectivity potential, although further studies are needed to confirm these predictions.

### 3.9. In Silico Pharmacokinetic Analysis

The physicochemical properties of compounds **AG-615**, **AG-410**, **CZ-018**, and **CZ-173** were predicted using the ADMETlab 2.0 website (Table 5). The ADME prediction study of the compounds provided the physicochemical properties of potential oral drug candidates according to Lipinski’s five rules [34,35]. The reference LogS value for soluble molecules ranged from −3.25 to −5.38. According to the results, almost all of the molecules are classified as soluble (except compound **CZ-173**). Another parameter considered important is drug interactions with cytochrome P450, which determines the elimination property through drug metabolism [35]. The analysis suggests that compounds **CZ-018** and **CZ-173** did not exhibit inhibitory activity against any of the tested isoforms, whereas compounds **AG-615** and **AG-410** showed inhibitory activity against the CYP2C9 isoform. Permeability glycoprotein (P-gp) is a key member of ATP-dependent membrane transporters, known as ABC transporters (ATP-binding cassette), and plays an essential role in efficient membrane transport and in protecting the central nervous system from toxic compounds [36,37]. Compounds **AG-615**, **AG-410**, and **CZ-018** are predicted substrates of P-gp.

To determine the toxicity of the selected compounds, the ProTox-II server testing tool was used (Table 5).

The software assessed liver toxicity and predicted the median lethal dose (LD_50_) (in mg/kg of body weight). According to the server, the compounds were predicted to have an LD_50_ of 750 to 5000 mg/kg, which is harmful when administered orally. Overall, the compounds showed a promising balance between pharmacodynamic and pharmacokinetic parameters, making them candidates for future evaluations.

Overall, the combined results of the bioassays and ADMET analysis indicate that **AG-410**, **AG-615**, **CZ-018**, and **CZ-173** possess favorable pharmacodynamic profiles that are characterized by potent trypanocidal activity, low cytotoxicity, and adequate selectivity. Furthermore, their predicted pharmacokinetic properties are consistent with those of early-stage oral drug candidates. Despite some anticipated limitations related to P-gp transport and CYP2C9 inhibition, the compounds, particularly **CZ-018** and **CZ-173**, demonstrate an acceptable balance for future in vivo studies to further evaluate their bioavailability, toxicity, and therapeutic potential.

## 4. Conclusions

In this study, a ligand-based virtual screening of FDA-approved drugs with activity against *T. cruzi* allowed the selection of thirteen compounds as potential *Tc*Cz inhibitors. A cefsulodin analog (LC_50_ = 126.18 and 77.50 µM); two flucloxacillin analogs (LC_50_ = 94.05 and 101.73 µM, and 48.74 and 64.49 µM); and a piperacillin analog (LC_50_ = 48.46 and 83.68 µM) had better trypanocidal activity against the NINOA and INC-5 strains than the reference drugs, benznidazole and nifurtimox. Furthermore, three of the compounds evaluated exhibited enzymatic inhibition of *T. cruzi* cysteine proteases and were predicted to be stable compounds with favorable selectivity and ADMET properties. These results, which are based on the structure of FDA-approved drugs, support the design and development of novel and more potent agents for the pharmacological treatment of Chagas disease.

## Figures and Tables

**Table 1:** Compounds obtained from the ligand-based virtual screening of the Molport database.

Criteria Applied	Scaffold Used			
	Etofylline Clofibrate	Cefsulodin Sodium	Flucloxacillin Sodium	Piperacillin Sodium
Structural similarity 50%	3306	10,000	2200	1547
Lipinski–Veber rules	2656	2142	778	907
BFE	2424	1927	743	864
Interaction analysis	211	188	74	110
Grouping by interactions	10	11	8	8
Control-like group	7	7	1	6
Compounds to acquire	**AG-287** **AG-405**	**AG-218** **AG-132** **AG-988** **AG-410**	**CZ-018** **AG-984** **AG-525** **CZ-899** **AG-615**	**CZ-173** **AG-595**

**Table 2:** Half-maximal lytic concentrations of the thirteen compounds selected via virtual screening against two *T. cruzi* strains.

Compound	*Trypanosoma cruzi* (µM)	
	NINOA	INC-5
**AG-988**	109.38 ± 2.19	>200
**AG-405**	>200	78.60 ± 2.27
**AG-287**	>200	>200
**AG-218**	99.90 ± 2.53	>200
**AG-410**	126.18 ± 4.17	77.50 ± 1.70
**AG-132**	>200	>200
**AG-615**	94.05 ± 1.95	101.73 ± 0.26
**AG-984**	>200	>200
**CZ-018**	48.74 ± 2.63	64.49 ± 4.34
**AG-525**	>200	>200
**CZ-899**	114.87 ± 4.10	>200
**AG-595**	25.54 ± 3.11	>200
**CZ-173**	48.46 ± 3.24	83.68 ± 3.08
**Benznidazole**	161.05 ± 1.08	254.93 ± 0.38
**Nifurtimox**	219.82 ± 0.58	317.85 ± 0.21

**Table 3:** Cytotoxic evaluation against the J774.2 macrophage cell line and SI of potential *Tc*Cz inhibitors.

Compound	Macrophage J774.2 CC_50_ (µM)	Selectivity Index *T. cruzi* Strain	
		NINOA	INC-5
**AG-410**	298.26 ± 0.29	2.36	3.84
**AG-615**	252.16 ± 0.05	2.68	2.47
**CZ-018**	306.08 ± 0.83	6.28	4.74
**CZ-173**	236.09 ± 0.87	4.87	2.82
**Benznidazole**	133.90 ± 0.06	0.83	0.52
**Nifurtimox**	164.20 ± 0.3	0.74	0.51

**Table 4:** Half-maximal inhibitory concentrations of potential *Tc*Cz inhibitors with trypanocidal activity against *T. cruzi* cysteine proteases.

Scaffold	Derivative	IC_50_ µM
Cefsulodin sodium	**AG-410**	120.97
Flucloxacillin sodium	**AG-615**	840.03
	**CZ-018**	85.03
Piperacillin sodium	**CZ-173**	44.16

**Table 5:** Prediction of the physicochemical and pharmacokinetic properties of compounds with trypanocidal activity.

Physicochemical and Pharmacokinetic Properties	Compounds			
	AG-615	AG-410	CZ-018	CZ-173
MW (g/mol) < 500	438.11	482.12	482.18	498.21
Rotatable bonds < 10	5	6	5	7
Hydrogen bond acceptors < 10	8	6	9	8
Hydrogen bond donors < 5	0	1	0	1
TPSA (Å^2^) < 140	98.4	77.89	87.83	91.84
Log *P* < 5	2.98	3.93	2.99	2.14
Log *S* −4–0.5 log mol/L	−4.65	−5.38	−4.89	−3.25
Permeability BBB	Yes	Yes	Yes	No
P-gp substrate	Yes	Yes	Yes	No
CYP1A2 inhibitor	Inactive	Inactive	Inactive	Inactive
CYP2C19 inhibitor	Inactive	Inactive	Inactive	Inactive
CYP2C9 inhibitor	Active	Active	Inactive	Inactive
CYP2D6 inhibitor	Inactive	Inactive	Inactive	Inactive
Hepatotoxicity	Inactive	Inactive	Inactive	Inactive
Predicted LD_50_ (mg/kg)	5000	900	5000	750

MW: molecular weight, TPSA: polar surface area, Log *P*: partition coefficient, Log *S*: solubility coefficient, and Permeability BBB: permeability of the blood–brain barrier.

## Data Availability

No data were used for the research described in the article.

## Supplementary Materials

The following supporting information can be downloaded at: https://www.mdpi.com/article/10.3390/diseases14020079/s1, Figure S1. Representation of the overlapped structure of the 3H5 control; Figure S2. Dose-response curve of compound AG-218 in the NINOA strain of *T. cruzi*; Figure S3. Dose-response curve of compound AG-218 in the INC-5 strain of *T. cruzi*; Figure S4. Dose-response curve of compound AG-218 in the INC-5 strain of *T. cruzi*; Figure S5. Dose-response curve of compound AG-405 in the INC-5 strain of *T. cruzi*; Figure S6. Dose-response curve of compound AG-615 in the NINOA strain of *T. cruzi*; Figure S7. Dose-response curve of compound AG-615 in the INC-5 strain of *T. cruzi*; Figure S8. Dose-response curve of compound AG-410 in the NINOA strain of *T. cruzi*; Figure S9. Dose-response curve of compound AG-410 in the INC-5 strain of *T. cruzi*; Figure S10. Dose-response curve of compound CZ-899 in the NINOA strain of *T. cruzi*; Figure S11. Dose-response curve of compound CZ-018 in the NINOA strain of *T. cruzi*; Figure S12. Dose-response curve of compound CZ-018 in the INC-5 strain of *T. cruzi*; Figure S13. Dose-response curve of compound AG-595 in the NINOA strain of *T. cruzi*; Figure S14. Dose-response curve of compound CZ-173 in the NINOA strain of *T. cruzi*; Figure S15. Dose-response curve of compound CZ-173 in the INC-5 strain of *T. cruzi*.

## Abbreviations

*T. cruzi*	*Trypanosoma cruzi*
CADD	Computer-Aided Drug Design
BFE	Binding free energy
MD	Molecular dynamics
RMSD	Root mean square deviation
RMSF	Root mean square fluctuation
Rgyr	Radius of gyration
PDB	Database Protein Data Bank
Cz	Cruzain

## References

[1] WHO Chagas Disease (American Trypanosomiasis). https://www.who.int/health-topics/chagas-disease#tab=tab_1.

[2] Rojo-Medina J., Ruiz-Matus C., Salazar-Schettino P.M., González-Roldán J.F. (2018). Chagas disease in Mexico. Gac. Med. Mex..

[3] Porcal W., Hernández P., Boiani L., Boiani M., Ferreira A., Chidichimo A., Cazzulo J.J., Olea-Azar C., González M., Cerecetto H. (2008). New trypanocidal hybrid compounds from the association of hydrazone moieties and benzofuroxan heterocycle. Bioorg. Med. Chem..

[4] Jackson Y., Wyssa B., Chappuis F. (2020). Tolerance to nifurtimox and benznidazole in adult patients with chronic Chagas’ disease. J. Antimicrob. Chemother..

[5] Nunes J.A., Santos-Júnior P.F.D.S., Gomes M.C., Ferreira L.A.S., Padilha E.K.A., Teixeira T.R., Stanger E.J., Kaur Y., Silva E.B.D., Costa C.A.C.B. (2025). Nanomolar activity of coumarin-3-thiosemicarbazones targeting *Trypanosoma cruzi* cruzain and the *T. brucei* cathepsin L-like protease. Eur. J. Med. Chem..

[6] Ripa J.D., Ali S., Field M., Smithson J., Wangchuk P. (2025). From AI-Assisted In Silico Computational Design to Preclinical In Vivo Models: A Multi-Platform Approach to Small Molecule Anti-IBD Drug Discovery. Pharmaceuticals.

[7] Palos I., Lara-Ramirez E.E., Lopez-Cedillo J.C., Garcia-Perez C., Kashif M., Bocanegra-Garcia V., Nogueda-Torres B., Rivera G. (2017). Repositioning FDA Drugs as Potential Cruzain Inhibitors from *Trypanosoma cruzi*: Virtual Screening, In Vitro and In Vivo Studies. Molecules.

[8] Pettersen E.F., Goddard T., Huang C.C., Couch G.S., Greenblatt D.M., Meng E.C., Ferrin T.E. (2004). UCSF Chimera—A visualization system for exploratory research and analysis. J. Comput. Chem..

[9] O’boyle N.M., Banck M., James C.A., Morley C., Vandermeersch T., Hutchison G.R. (2011). Open Babel: An open chemical toolbox. J. Cheminform..

[10] McNutt A.T., Francoeur P., Aggarwal R., Masuda T., Meli R., Ragoza M., Sunseri J., Koes D.R. (2021). GNINA 1.0: Molecular docking with deep learning. J. Cheminform..

[11] Adasme M.F., Linnemann K.L., Bolz S.N., Kaiser F., Salentin S., Haupt V.J., Schroeder M. (2021). PLIP 2021: Expanding the scope of the protein-ligand interaction profiler to DNA and RNA. Nucleic Acids Res..

[12] Pedregosa F., Varoquaux G., Gramfort A., Michel V., Thirion B., Grisel O., Blondel M., Prettenhofer P., Weiss R., Dubourg V. (2011). Scikit-learn: Machine Learning in Python. J. Mach. Learn. Res..

[13] Chacón-Vargas K.F., Nogueda-Torres B., Sánchez-Torres L.E., Suarez-Contreras E., Villalobos-Rocha J.C., Torres-Martinez Y., Lara-Ramírez E.E., Fiorani G., Krauth-Siegel R.L., Bolognesi M.L. (2017). Trypanocidal Activity of Quinoxaline 1,4 Di-*N*-oxide Derivatives as Trypanothione Reductase Inhibitors. Molecules.

[14] Delgado-Maldonado T., Moreno-Rodriguez A., Gonzalez-Morales L.D., Florez-Villegas A.L., Rodriguez-Gonzalez J., Rodriguez-Paez L., Aguirre-Alvarado C., Sanchez-Palestino L.M., Ortíz-Pérez E., Rivera G. (2024). Design, Synthesis, and In Vitro and In Silico Evaluation of 1,3,4-Oxadiazoles as Anti-*Trypanosoma cruzi* and *Anti-Leishmania mexicana* Agents. Chem. Med. Chem..

[15] Bradford M.M. (1976). A Rapid and Sensitive Method for the Quantitation of Microgram Quantities of Protein Utilizing the Principle of Protein-Dye Binding. Anal. Biochem..

[16] Santos C.C., Sant’anna C., Terres A., Cunha-e-Silva N.L., Scharfstein J., de A. Lima A.P. (2005). Chagasin, the Endogenous Cysteine-Protease Inhibitor of Trypanosoma cruzi, Modulates Parasite Differentiation and Invasion of Mammalian Cells. J. Cell Sci..

[17] Ritz C., Baty F., Streibig J.C., Gerhard D. (2015). Dose-Response Analysis Using R. PLoS ONE.

[18] Abraham M.J., Murtola T., Schulz R., Páll S., Smith J.C., Hess B., Lindah E. (2015). GROMACS: High performance molecular simulations through multi-level parallelism from laptops to supercomputers. SoftwareX.

[19] Polishchuk P., Kutlushina A., Bashirova D., Mokshyna O., Madzhidov T. (2019). Virtual Screening Using Pharmacophore Models Retrieved from Molecular Dynamic Simulations. Int. J. Mol. Sci..

[20] Lemkul J.A. (2019). From Proteins to Perturbed Hamiltonians: A Suite of Tutorials for the GROMACS-2018 Molecular Simulation Package [Article v1.0]. Living J. Comput. Mol. Sci..

[21] Wang J., Wang W., Kollman P.A., Case D.A. (2006). Case, Automatic atom type and bond type perception in molecular mechanical calculations. J. Mol. Graph. Model..

[22] Xiong G., Wu Z., Yi J., Fu L., Yang Z., Hsieh C., Yin M., Zeng X., Wu C., Lu A. (2021). ADMETlab 2.0: An integrated online platform for accurate and comprehensive predictions of ADMET properties. Nucleic. Acids. Res..

[23] Banerjee P., Eckert A.O., Schrey A.K., Preissner R. (2018). ProTox-II: A webserver for the prediction of toxicity of chemicals. ProTox-II: A webserver for the prediction of toxicity of chemicals. Nucleic Acids Res..

[24] Ounthaisong U., Tangyuenyongwatana P. (2017). Cross-docking study of flavonoids against tyrosinase enzymes using PyRx 0.8 virtual screening tool. Thai J. Pharm. Sci..

[25] Mateev E., Valkova I., Angelov B., Georgieva M., Zlatkov A. (2022). Validation through re-docking, cross-docking and ligand enrichment in various well-resoluted MAO-B receptors. Int. J. Pharm. Sci. Res..

[26] Brinen L.S., Hansell E., Cheng J., Roush W.R., McKerrow J.H., Fletterick R.J. (2000). A target within the target: Probing cruzain’s P1′ site to define structural determinants for the Chagas’ disease protease. Struct. Lond. Engl..

[27] Herrera-Mayorga V., Lara-Ramírez E.E., Chacón-Vargas K.F., Aguirre-Alvarado C., Rodríguez-Páez L., Alcántara-Farfán V., Cordero-Martínez J., Nogueda-Torres B., Reyes-Espinosa F., Bocanegra-García V. (2019). Structure-Based Virtual Screening and In Vitro Evaluation of New *Trypanosoma cruzi* Cruzain Inhibitors. Int. J. Mol. Sci..

[28] Bellera C.L., Balcazar D.E., Alberca L., Labriola C.A., Talevi A., Carrillo C. (2014). Identification of levothyroxine antichagasic activity through computer-aided drug repurposing. Sci. World J..

[29] Bellera C.L., Balcazar D.E., Vanrell M.C., Casassa A.F., Palestro P.H., Gavernet L., Labriola C.A., Gálvez J., Bruno-Blanch L.E., Romano P.S. (2015). Computer-guided drug repurposing: Identification of trypanocidal activity of clofazimine, benidipine and saquinavir. Eur. J. Med. Chem..

[30] Silva L.R., Guimarães A.S., do Nascimento J., do Santos Nascimento I.J., da Silva E.B., McKerrow J.H., Cardoso S.H., da Silva-Júnior E.F. (2021). Computer-aided design of 1,4-naphthoquinone-based inhibitors targeting cruzain and rhodesain cysteine proteases. Bioorg. Med. Chem..

[31] Bhowmik D., Jagadeesan R., Rai P., Nandi R., Gugan K., Kumar D.J. (2021). Evaluation of potential drugs against leishmaniasis targeting catalytic subunit of *Leishmania donovani* nuclear DNA primase using ligand based virtual screening, docking and molecular dynamics approaches. J. Biomol. Struct. Dyn..

[32] Kumari M., Subbarao N. (2020). Virtual screening to identify novel potential inhibitors for Glutamine synthetase of *Mycobacterium tuberculosis*. J. Biomol. Struct. Dyn..

[33] Méndez-Álvarez D., Herrera-Mayorga V., Juárez-Saldivar A., Paz-González A.D., Ortiz-Pérez E., Bandyopadhyay D., Pérez-Sánchez H., Rivera G. (2022). Ligand-based virtual screening, molecular docking, and molecular dynamics of eugenol analogs as potential acetylcholinesterase inhibitors with biological activity against *Spodoptera frugiperda*. Mol. Divers..

[34] Lipinski C.A., Lombardo F., Dominy B.W., Feeney P.J. (2001). Experimental and computational approaches to estimate solubility and permeability in drug discovery and development settings. Adv. Drug Deliv. Rev..

[35] Benet L.Z., Hosey C.M., Ursu O., Oprea T.I. (2016). BDDCS, the Rule of 5 and drugability. Adv. Drug Deliv. Rev..

[36] Rathod S.B., Prajapati P.B., Pal R., Mansuri M.F. (2023). AMPA GluA2 subunit competitive inhibitors for PICK1 PDZ domain: Pharmacophore-based virtual screening, molecular docking, molecular dynamics simulation, and ADME studies. J. Biomol. Struct. Dyn..

[37] Szakács G., Váradi A., Özvegy-Laczka C., Sarkadi B. (2008). The role of ABC transporters in drug absorption, distribution, metabolism, excretion and toxicity (ADME-Tox). Drug Discov. Today.

