# Supplementary material for: Identification of Novel Trypanosoma cruzi Cysteine Protease Inhibitors via Ligand-Based Virtual Screening of FDA-Approved Drugs with Trypanocidal Activity
Source: Diseases. 2026 Feb 19;14(2):79. doi: 10.3390/diseases14020079 (PMC12939925; doi:10.3390/diseases14020079)
Supplement: Supplementary file 1 [file diseases-14-00079-s001.zip › diseases-4126314-supplementary.pdf]

Identification of Novel *Trypanosoma cruzi* Cysteine Protease Inhibitors via Ligand-Based Virtual Screening of FDA-Approved Drugs with Trypanocidal Activity

Lenci K. Vázquez-Jiménez [1,2], Alonzo González-González [1], Timoteo Delgado-Maldonado [1], Rogelio Gómez-Escobedo [3], Benjamín Nogueda-Torres [3], Ana Verónica Martinez-Vazquez [1], Eyrá Ortiz-Pérez [1], Charmina Aguirre-Alvarado [4], Verónica Alcántara-Farfán [4], Joaquín Cordero-Martínez [4], Lorena Rodríguez-Páez [4] Adriana Moreno-Rodriguez[5] and Gildardo Rivera [1,*]

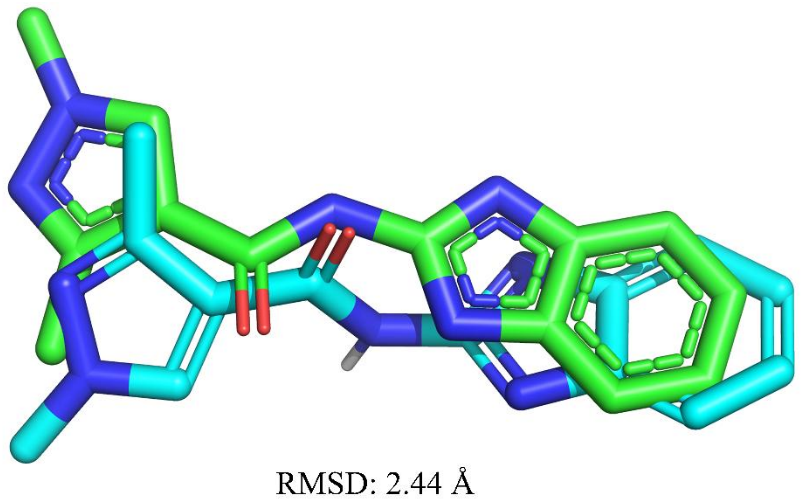

Figure S1. Representation of the overlapped structure of the 3H5 control (green) and the coupled pose (cyan). The RMSD between the two molecules is 2.44 Å.

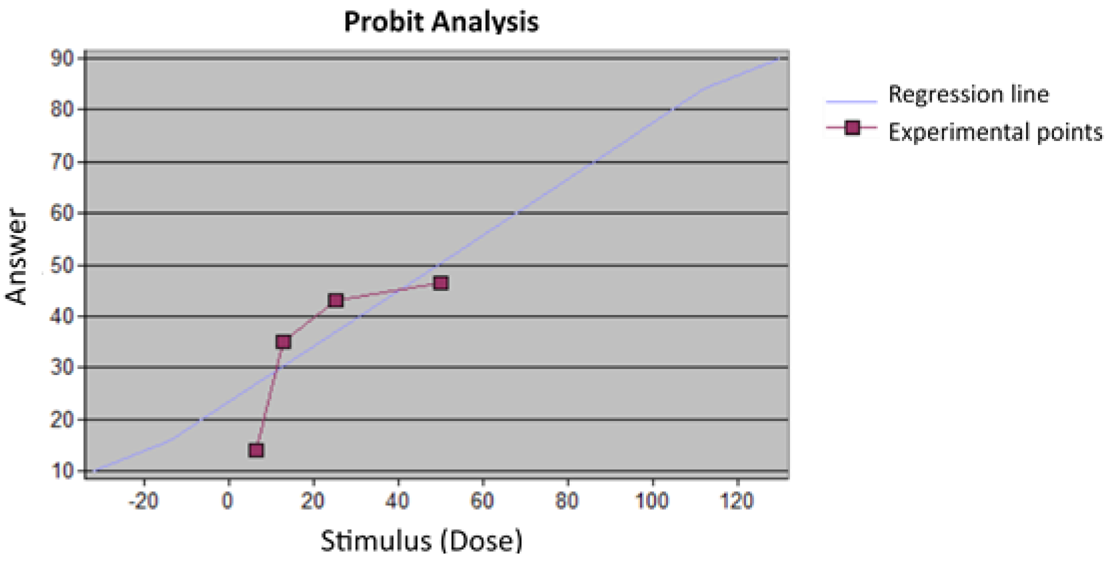

Figure S2. Dose-response curve of compound AG-218 in the NINOA strain of *T. cruzi*.

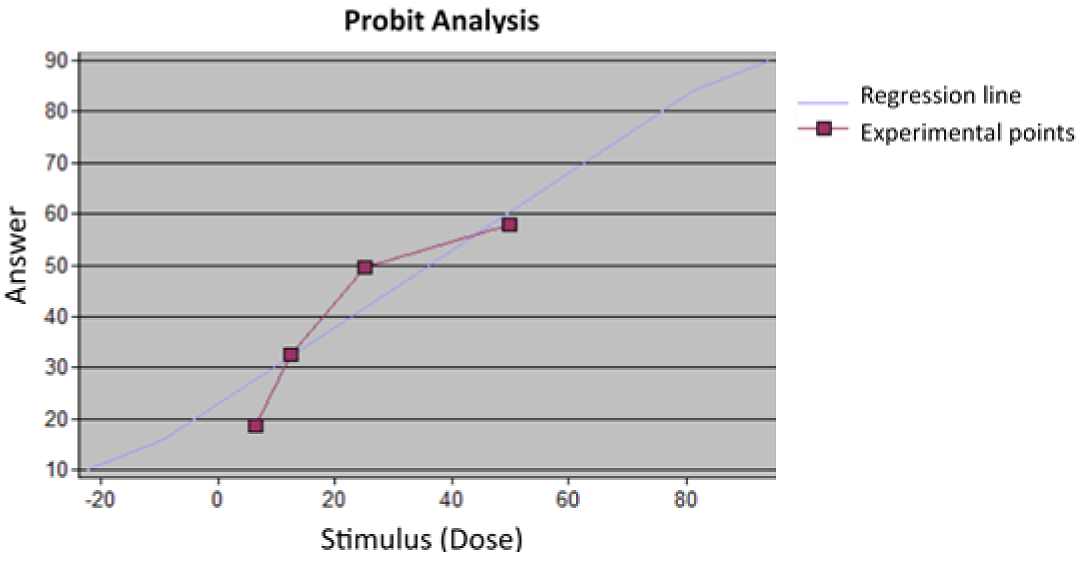

Figure S3. Dose-response curve of compound AG-218 in the INC-5 strain of *T. cruzi*.

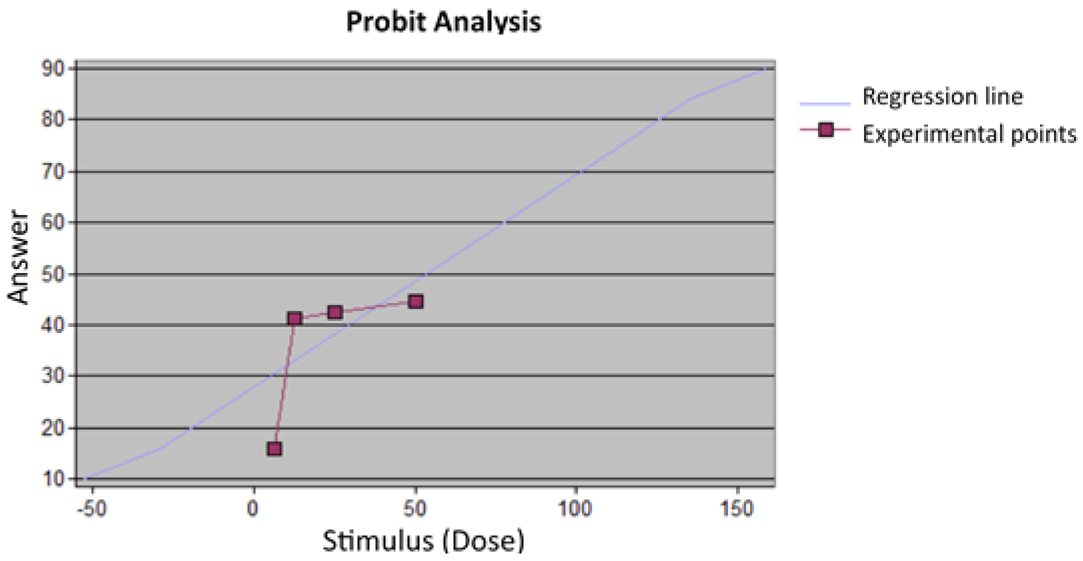

Figure S4. Dose-response curve of compound AG-988 in the NINOA strain of *T. cruzi*.

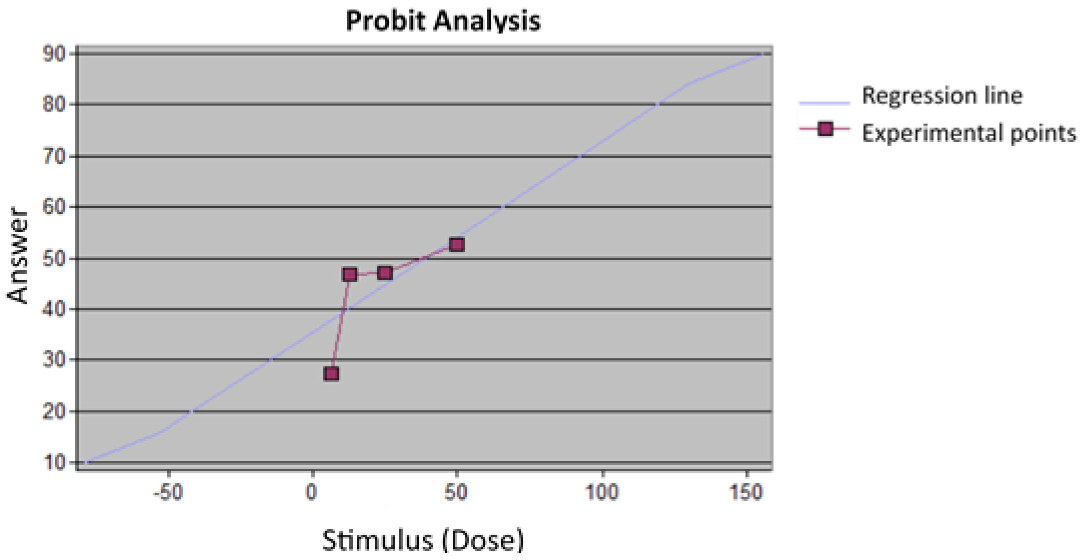

Figure S5. Dose-response curve of compound AG-405 in the INC-5 strain of *T. cruzi*.

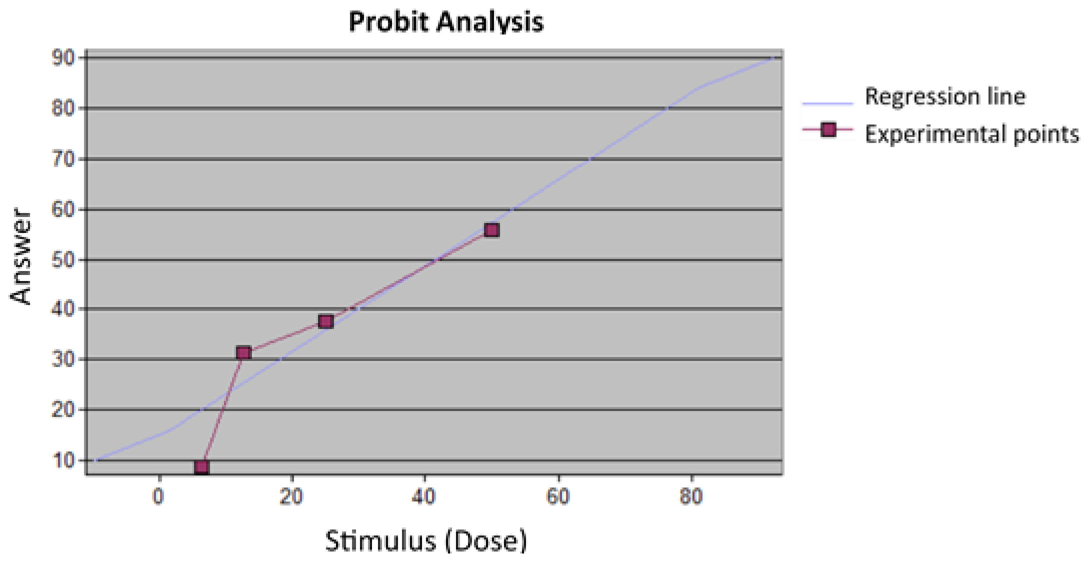

Figure S6. Dose-response curve of compound AG-615 in the NINOA strain of *T. cruzi*.

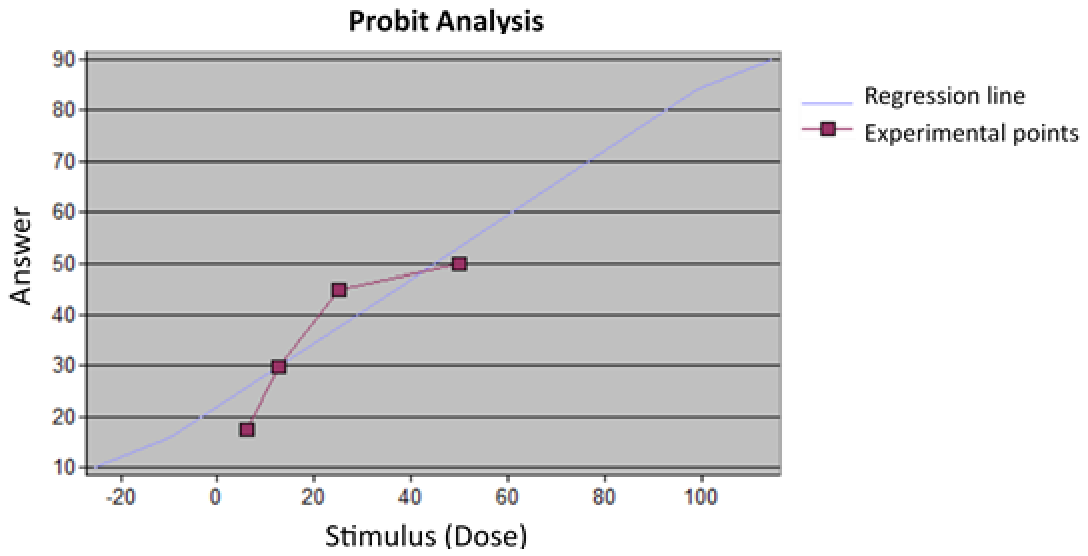

Figure S7. Dose-response curve of compound AG-615 in the INC-5 strain of *T. cruzi*.

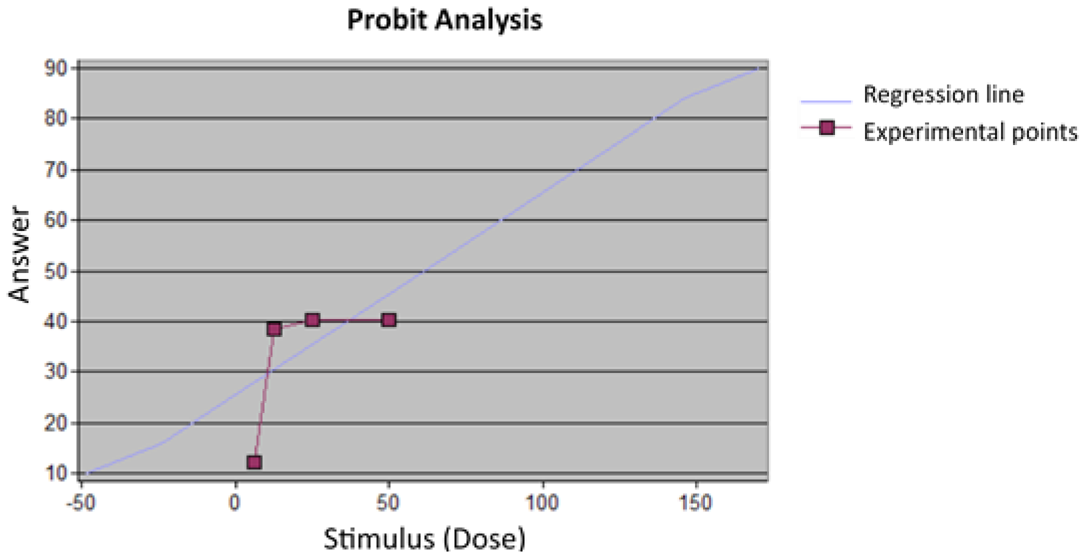

Figure S8. Dose-response curve of compound AG-410 in the NINOA strain of *T. cruzi*.

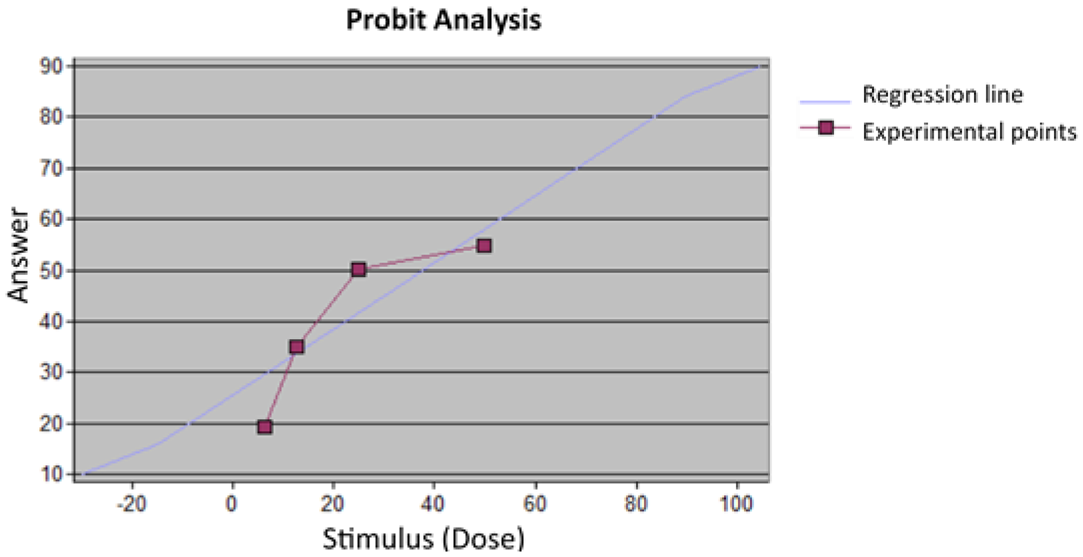

Figure S9. Dose-response curve of compound AG-410 in the INC-5 strain of *T. cruzi*.

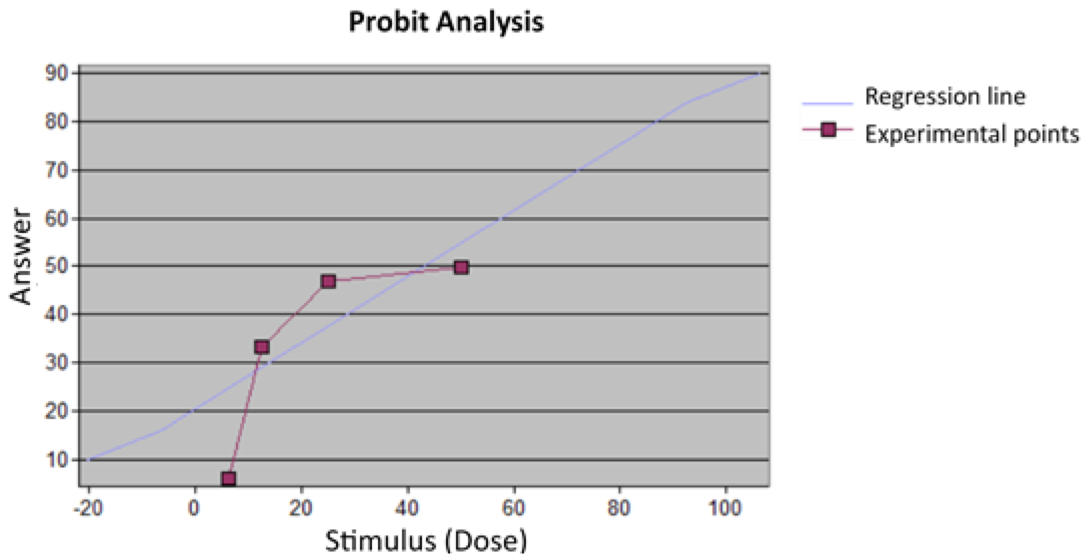

Figure S10. Dose-response curve of compound CZ-899 in the NINOA strain of *T. cruzi*.

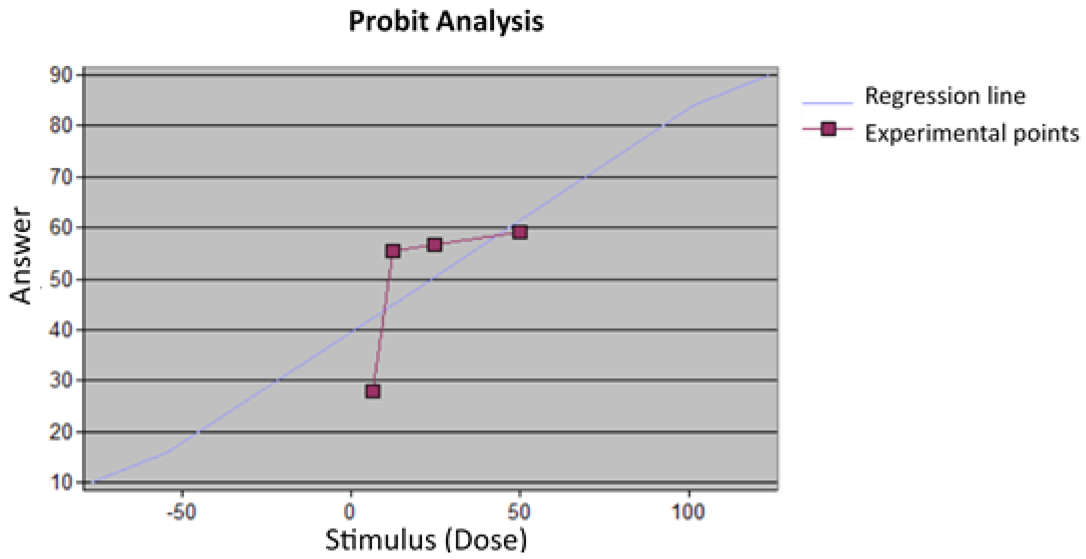

Figure S11. Dose-response curve of compound CZ-018 in the NINOA strain of *T. cruzi*.

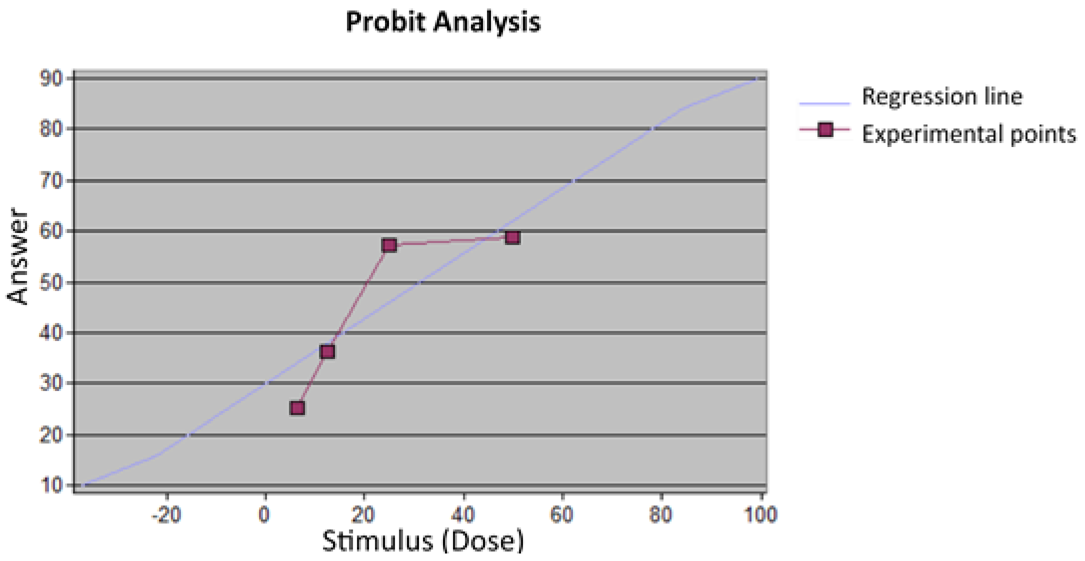

Figure S12. Dose-response curve of compound CZ-018 in the INC-5 strain of *T. cruzi*.

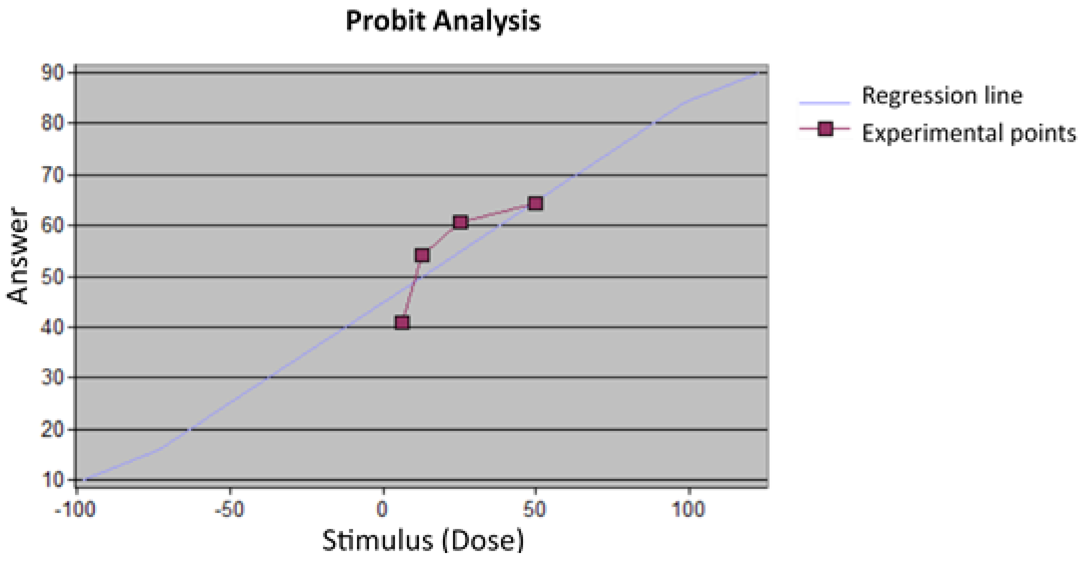

Figure S13. Dose-response curve of compound AG-595 in the NINOA strain of *T. cruzi*.

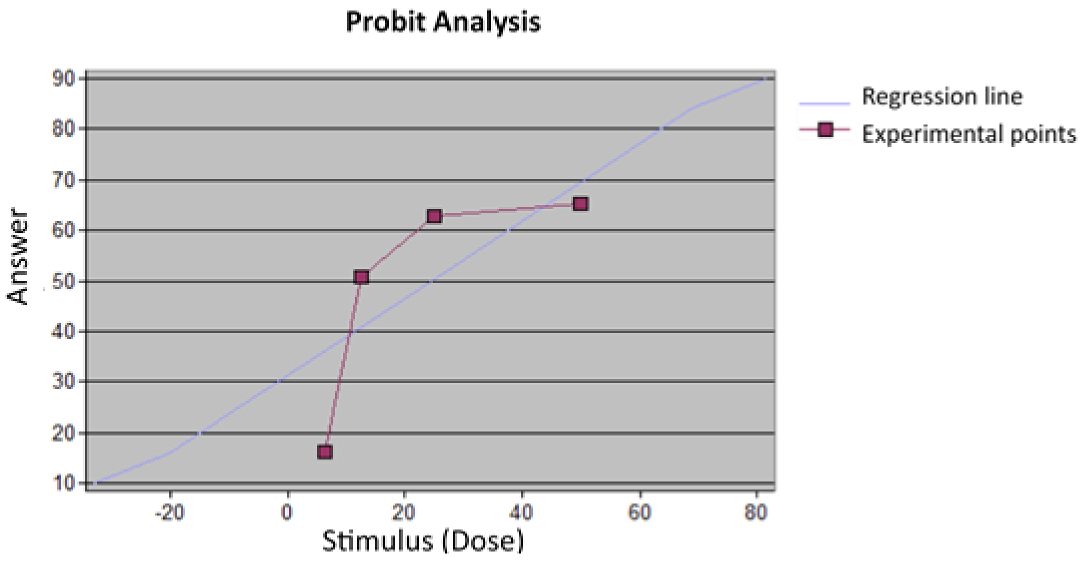

Figure S14. Dose-response curve of compound CZ-173 in the NINOA strain of *T. cruzi*.

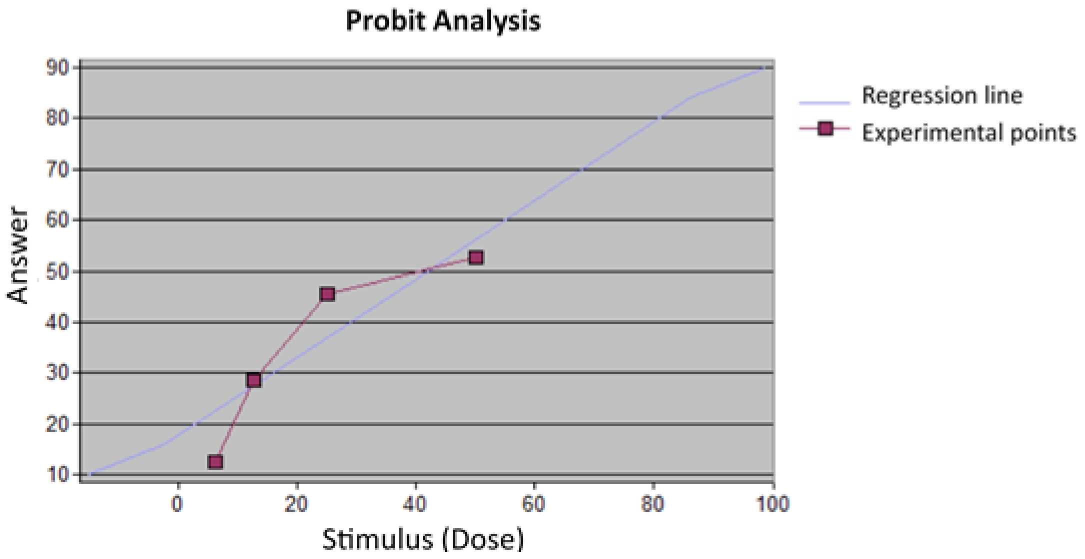

Figure S15. Dose-response curve of compound CZ-173 in the INC-5 strain of *T. cruzi*.
